# Phantom studies in medical imaging (PSMI): a guide with recommendations and checklist

**DOI:** 10.1186/s41747-025-00641-7

**Published:** 2025-11-03

**Authors:** Gisella Gennaro, Moreno Zanardo, Federico Ambrogi, Francesco Sardanelli

**Affiliations:** 1 Veneto Institute of Oncology (IOV), IRCCS, Padua, Italy; 2 Radiology Unit, IRCCS Policlinico San Donato, San Donato Milanese, Italy; 3 Dipartimento di Scienze Cliniche e di Comunità, Università degli Studi di Milano, Milano, Italy; 4 Laboratory of Biostatistics and Data Management, IRCCS Policlinico San Donato, San Donato Milanese, Italy; 5 Lega Italiana per la Lotta contro i Tumori (LILT) Milano Monza Brianza, Milan, Italy

**Keywords:** Diagnostic imaging, Methods, Phantoms (imaging), Reproducibility of results, Research design

## Abstract

**Abstract:**

Phantom studies are essential in medical imaging, offering a controlled and reproducible framework for evaluating imaging technologies across all modalities. Phantoms, whether physical (synthetic, biological, or mixed) or computational, simulate human tissues or anatomical structures and serve roles in technology validation, performance benchmarking, protocol optimization, quality assurance, and artificial intelligence development. We provide recommendations for designing and conducting phantom studies in medical imaging (PSMI). Key aspects include phantom selection, image acquisition protocols, and analysis strategies, particularly when image quality is evaluated in relation to radiation dose or contrast agent optimization. Quantitative image analysis is considered with emphasis on signal-to-noise ratio, contrast-to-noise ratio, and spatial resolution (*e.g*., modulation transfer function). Qualitative assessment is addressed considering reader selection and training, blinding, randomization, and use of absolute or relative Likert scales. Brief recommendations for sample size calculation, data reporting, and statistical analysis are provided, covering continuous/ordinal data, inter-rater agreement, and group comparisons. A checklist is provided to allow authors to document adherence to these recommendations and to identify shortcomings, limitations, and weaknesses in their phantom studies. The PSMI checklist is proposed to promote transparency, reproducibility, and critical appraisal, containing 25 items regarding: title/abstract (1, 2); background/introduction (3); methods/study design (4); methods/phantom description (5–7); methods/imaging protocol (8, 9); methods/image analysis (10, 11); methods/statistics (12–15); results/quantitative analysis (16, 17); results/qualitative analysis (18); results/tables and figures (19); discussion (20–23); and conclusions (24, 25). Finally, the importance of maintaining a clinical perspective is underscored, highlighting how well-designed phantom studies can inform, but not replace, clinical validation.

**Relevance statement:**

This paper provides comprehensive recommendations for designing and conducting PSMI. The use of the PSMI checklist may contribute to increasing the quality of phantom studies.

**Key Points:**

- Phantom studies provide controlled, reproducible evaluation of imaging technologies.
- Phantoms simulate human tissues for validation, optimization, and AI development.
- Good design includes proper phantom selection and analysis strategies.
- Clinical relevance must guide interpretation; phantoms cannot replace clinical validation.
- The proposed 25-item PSMI checklist supports transparent and reproducible phantom study reporting.

**Graphical Abstract:**

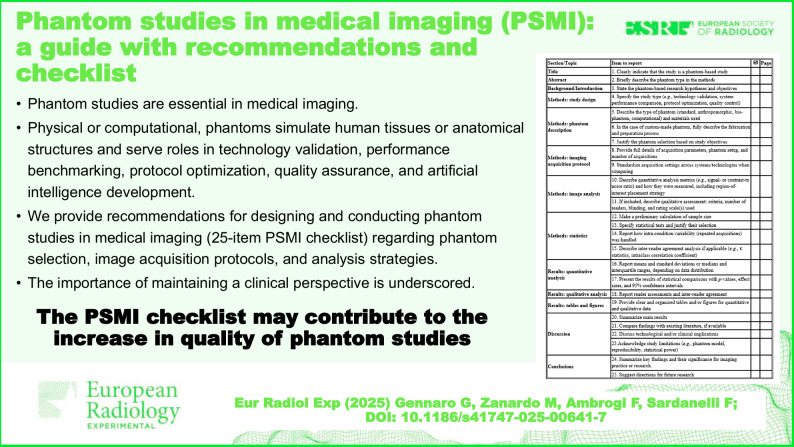

## Background

Phantom studies play a critical role in medical imaging by providing a controlled and reproducible environment for evaluating imaging systems, testing algorithms, and optimizing clinical workflows. Phantoms are physical or computational models designed to simulate human tissues or anatomical structures. Their utility spans all imaging modalities, including conventional radiography, mammography, computed tomography (CT), magnetic resonance imaging (MRI), and ultrasound. They are also extensively used in nuclear medicine techniques such as positron emission tomography and single-photon emission CT, as well as in radiation therapy for both treatment planning, quality assurance, and dose verification [1].

While the methodological principles discussed in this article are broadly applicable, the focus is limited to phantom studies in diagnostic radiology. Within this framework, phantom studies remain indispensable tools to help researchers to technically evaluate imaging modalities [2], develop new imaging techniques [3, 4], and validate image analysis methods [5]. Notably, these tasks can be accomplished without the ethical concerns typically associated with human or animal studies. Consequently, phantom studies do not require approval from an ethics committee [6]; however, a general ethical concern should always be considered for the economic cost and work used for the experiments, as well as the equipment work time, with potential delay of clinical use.

Phantom studies serve multiple critical applications across different imaging modalities. They are essential for *quality control and assurance*, ensuring that systems maintain consistent performance over time by evaluating factors such as resolution, contrast, noise, and radiation dose [7–10]. They also play a key role in *protocols optimization*, allowing researchers to test and refine acquisition and reconstruction parameters under controlled conditions before applying them in clinical practice [11–13]. Moreover, phantom studies *facilitate comparative analysis of imaging technologies*, enabling direct performance assessments of imaging modalities, scanners, or reconstruction algorithms without patient-related variability [14–16]. Finally, in recent years, they have become increasingly important in the development and validation of artificial intelligence (AI) applications in medical imaging, providing datasets with known ground truth for training and testing AI models in image processing, segmentation, and quantitative analysis [17–20].

Despite these objective advantages, phantom studies, if poorly designed, can lead to misleading conclusions. Sometimes, reading a phantom study, we have the impression that there was no real experimental design, with a well-defined rationale: a large number of measurements have been collected without a structured plan, and the paper was assembled retrospectively to fit the data. Issues such as unclear study objectives, inadequate phantom design, lack of reproducibility evaluation, few readers for qualitative image quality assessment, inappropriate rating scales and/or statistical methods, and failure to consider clinical relevance are common pitfalls that undermine the validity of findings in phantom studies [21–23].

Here, we provide recommendations for designing and conducting robust phantom studies in medical imaging (PSMI), outlining key considerations in phantom selection, imaging protocols, image analysis methods, and statistical approaches. By addressing common limitations and best practices, we aim to improve the clarity, reproducibility, and scientific value of phantom-based research, and ensure that these studies effectively contribute to the development and validation of imaging technologies. A 25-item checklist is provided to allow authors to document adherence to these recommendations and to identify shortcomings, limitations, and weaknesses in their phantom studies.

## Objectives of phantom studies

Before designing a phantom study, it is essential to clearly define its objectives, *i.e.*, to have a precise scientific question or hypothesis, ensuring that all subsequent methodological choices align with this goal. Given that phantom studies allow for multiple acquisitions under controlled conditions, they can often address more than one objective. However, these objectives must be explicitly stated and consistently maintained throughout the manuscript, from the abstract’ background/purposes to the conclusions of the body text, to avoid ambiguity, and —most importantly—to avoid “*p*-hacking” after finishing the measurements [24, 25].

Common objectives in phantom studies include:

- technology validation = assessing the performance of new imaging modalities or testing novel technologies and algorithms in established imaging techniques;
- system performance comparison = comparing different imaging equipment or pieces of equipment, using quantitative and/or qualitative image quality assessment methods;
- protocol optimization = comparing different acquisition and/or reconstruction settings to identify the best applied in an effective clinical protocol;
- quality control = measuring physical parameters such as spatial resolution, contrast, noise, dose efficiency, or other quantitative image quality metrics to ensure system reliability and compliance with standards.

Table 1 provides examples of well-defined study purposes and common pitfalls in poorly structured phantom research. Clearly stated objectives contribute to a coherent study design, ensuring that the methodology, data collection, and analysis are structured to provide meaningful and reproducible results.

**Table 1:** Examples of good and bad definitions of study purpose in phantom studies

Purpose category	Good Example	Bad Example
Technology validation	“This study aims to evaluate the accuracy of a new deep-learning-based reconstruction algorithm in reducing noise in low-dose CT images using a standard phantom.”	*“This study aims to evaluate the potential of AI-based reconstruction methods to improve clinical image quality.”*
System performance comparison	“This study aims to compare the CNR and spatial resolution of PC-CT and EID-CT using a multi-material phantom. In addition, a qualitative image quality assessment will be performed through a blinded reader study to evaluate lesion conspicuity and perceived noise levels.”	*“This study aims to evaluate the superiority of photon-counting CT over conventional CT through image analysis and expert review.”*
Protocol optimization	“This study aims to compare the impact of different MRI acquisition parameters on lesion detectability in a dedicated anthropomorphic breast phantom.”	*“This study aims to define the best MRI protocol for breast imaging.”*
Quality control	“This study aims to quantify image noise and uniformity across multiple CT scanners using a homogeneous phantom and standardized acquisition parameters.”	*“This study aims to check image quality in different CT scanners.”*

*AI* Artificial intelligence, *CNR* Contrast-to-noise ratio, *EID-CT* Energy-integrating detector CT, *CT* Computed tomography, *MRI* Magnetic resonance imaging, *PC-CT* Photon-counting CT

## Phantoms classification

The selection of an appropriate phantom is a critical step in the design of a phantom study, as it has a direct impact on the relevance, reliability, and reproducibility of the results. Phantoms are used to simulate or approximate human anatomy and tissue properties under controlled conditions to evaluate and optimize imaging systems, protocols, and technologies. They can be broadly classified into physical and computational phantoms, each serving a different purpose depending on the study objectives, with further subclassification for the first group Table 2.

**Table 2:** Classification of phantoms used in medical imaging research

Physical phantoms	Synthetic	Standard (*e.g*., PMMA, solid-water)
		Anthropomorphic (*e.g*., 3D-printed models)
	Biological (biophantom, *e.g*., part of animals, vegetables)	
	Mixed (*e.g*., biological inserts embedded into synthetic phantoms)	
Computational phantoms (*e.g*., Monte Carlo simulations, finite element modeling, AI)		

*3D* Three-dimensional, *AI* Artificial intelligence, *PMMA* Polymethyl methacrylate

### Physical phantoms

Physical phantoms are tangible objects designed to mimic the physical properties of human tissues for specific imaging modalities (*e.g*., x-ray attenuation, ultrasound attenuation, or MRI relaxation properties). They can be divided into three main categories, which we refer to as standard phantoms, anthropomorphic phantoms, mixed phantoms, and biological phantoms (biophantoms). In particular, because standard and anthropomorphic phantoms are made entirely of non-biological materials, we collectively refer to them as synthetic phantoms.

#### Standard synthetic phantoms

Standard phantoms are constructed from simple, well-characterized materials, making them ideal for evaluating basic imaging system parameters such as resolution, contrast, noise, and geometric accuracy. These phantoms are typically made of durable materials such as polymethylmethacrylate, solid water, or other compounds, chosen based on the imaging modality and the parameters being tested. Solid water phantoms are commonly used in CT imaging due to their tissue-equivalent x-ray attenuation properties [26], and polymethylmethacrylate phantoms are frequently employed in x-ray and mammography [27]. Ultrasound and MRI imaging phantoms often incorporate gels or rubber-based materials to simulate acoustic and nuclear magnetic relaxation properties, respectively [28–30].

Standard phantoms play a role in calibrating imaging systems, assessing image quality, and optimizing system components such as automatic exposure control in x-ray imaging [11, 31]. When used for image quality evaluation, they must accurately reproduce attenuation, electronic or proton^1^ density, or elasticity similar to human tissues. For example, a CT quality assessment phantom should provide a realistic x-ray attenuation, appropriate material compositions to mimic different tissues, and contrast levels sufficiently similar to those of human tissues.

#### Anthropomorphic synthetic phantoms

Anthropomorphic phantoms are designed to replicate human anatomy and tissue heterogeneity, providing a closer approximation to clinical conditions than that allowed by standard synthetic phantoms. These phantoms are particularly valuable for testing and optimizing imaging protocols intended for clinical use, evaluating image reconstruction techniques, and validating advanced technologies such as AI-based diagnostic algorithms under near-real conditions. Anthropomorphic phantoms are constructed using tissue-equivalent materials, such as polymers, silicones, or composite materials, designed to match the x-ray attenuation, electronic/proton density, or elasticity of human tissues. They may be commercially produced [32, 33] or custom-built through mechanical manufacturing methods or three-dimensional (3D) printing technologies to enhance anatomical accuracy [34–36].

#### Mixed phantoms

Mixed phantoms combine synthetic structures with biological tissues to capture both the anatomical fidelity of synthetic components and the realistic imaging characteristics of biological materials. For example, excised human or animal tissues (*e.g.*, formalin-fixed samples from cadavers or veterinary specimens) can be embedded within an anthropomorphic shell to replicate the global attenuation and scattering properties of the human body while maintaining internal structures that closely mimic real tissue. This approach is particularly useful when the imaging task requires realistic texture, microstructure, or contrast kinetics that are difficult to replicate synthetically.

Such designs are valuable for repeated acquisitions, provided that appropriate preservation methods (*e.g.*, formalin fixation) are used. However, challenges include tissue preservation, potential changes in imaging characteristics over time, ethical considerations, and biosafety requirements. All preparation, storage, and handling procedures must be clearly documented to ensure reproducibility and safety.

#### Biophantoms

Biophantoms are phantoms made entirely from biological materials that approximate the physical, chemical, and structural properties of human tissues more closely than synthetic phantoms. These include animal-based phantoms, such as excised organs, muscles, or bones from pigs or other species, which are commonly used in ultrasound, CT, and MRI studies, and plant-based phantoms, such as agar, gelatin, or plant tissues, which have been investigated for their ability to mimic soft tissue properties in certain imaging modalities. Sometimes even common vegetables such as kiwifruit, potatoes, or oranges have been used as phantom surrogates [37, 38].

While bio-phantoms can successfully achieve similarity or equivalence to human tissue in terms of composition and structure for some types of imaging, they present significant challenges in terms of preservation, consistency, and reproducibility over time. Their natural variability in density, moisture content, and structural integrity can lead to measurement inconsistencies that make standardization difficult, and their degradation over time prevents even the experiment performed at a specific site from being reproducible [39, 40].

#### Physical phantoms: reproducibility of results

The choice of phantom has a direct impact on the reproducibility and generalizability of study results. *Commercially available synthetic and mixed phantoms*, manufactured under controlled processes, generally provide higher reproducibility [41], making them preferable for comparative studies, quality control, and regulatory compliance. However, they can be expensive and less adaptable to highly specialized research needs.

*Homemade synthetic and mixed phantoms* offer greater flexibility but suffer from a lack of standardization and quality control, making them more difficult to reproduce. For example, in CT studies on contrast media optimization, it is insufficient to simply place vials containing different contrast concentrations in the imaging field without replicating the overall attenuation of the human body. In mixed designs, the integration of biological tissues must also account for potential changes over time, ensuring that results remain comparable across acquisitions.

Finally, bio-phantoms might offer good tissue replication. However, they have significant limitations due to high variability, limited stability over time, and poor reproducibility, making them unsuitable for controlled experimental designs. Their use should be limited to proof-of-concept studies where precise standardization is not a critical requirement. In addition, the use of common vegetables should be discouraged and ultimately limited to interventional models where the primary goal is to reproduce the geometric characteristics of the organ undergoing intervention (*e.g*., size and shape of a prostate) rather than to assess image quality, as their imaging properties are generally not representative of human tissues.

Since reproducibility is a cornerstone of scientific validity, physical phantom-based experiments must be designed to ensure that other researchers can reproduce the results under the same conditions. If a homemade, mixed, or bio-phantom is used because there is no other choice, the phantom fabrication process, material properties, and preparation details must be fully documented in the main text of the manuscript (in the “Methods” section) and not relegated to supplementary materials.

### Computational phantoms

Computational phantoms are digital models that simulate human anatomy and tissue properties for use in virtual imaging experiments. They are particularly valuable in scenarios where physical phantom fabrication is impractical or where large parameter variations are required. They range from simple geometric shapes used for system calibration [42] to highly detailed anatomical models derived from medical imaging data [43]. They enable simulation-based studies to evaluate imaging system performance, optimize acquisition and reconstruction parameters, and develop new imaging techniques without the need for repeated physical acquisitions.

A unique advantage of computational phantoms is the ability to estimate radiation dose to specific anatomical locations with high spatial precision. something that is difficult to achieve with physical phantoms unless invasive measurement devices are used, which may alter imaging conditions. Computational methods allow this type of analysis without introducing foreign objects that could affect image formation.

Monte Carlo simulations, finite element modeling, and AI-driven phantoms are commonly used to study dose distribution, imaging processes, and artifact behavior in a fully controlled and reproducible environment. Unlike physical phantoms, computational phantoms offer perfect repeatability and allow systematic studies that would be impossible or unethical in real-world conditions. However, their effectiveness depends on how accurately they model tissue properties and imaging physics. Validation against experimental or clinical data is required to ensure their reliability [44].

## Image acquisition protocols

A well-defined image acquisition protocol is essential to ensure the reliability and reproducibility of physical phantom studies. The acquisition protocol must be tailored to the objectives of the study, whether it involves characterizing an imaging system, comparing different imaging systems or technologies, or optimizing image quality relative to limiting factors such as radiation dose in x-ray-based techniques, contrast agent dose in contrast-enhanced modalities, or acquisition time and number of sequences in MRI. Each of these scenarios requires specific considerations to minimize bias, ensure consistency, and allow for meaningful comparisons.

Table 3 summarizes key acquisition protocol considerations for different study types.

**Table 3:** Study objectives and corresponding acquisition protocol considerations

Study type	Purpose	Key acquisition considerations
Imaging system characterization	Evaluate the performance of a single imaging system under different conditions or in specific conditions	→ Systematic variation of acquisition parameters or reconstruction algorithms → Multiple acquisitions to evaluate reproducibility
Comparison between systems	Compare image quality or dose performance across different imaging systems of the same technology	→ Standardized phantom positioning, acquisition settings, and processing across systems → Cross-calibration to account for system-specific differences → Multiple acquisitions to evaluate reproducibility
Comparison between imaging technologies	Assess differences between different technologies	→ Define equivalence criteria for image quality metrics → Address differences in inherent system design → Multiple acquisitions to evaluate reproducibility
Image quality *versus* dose/contrast optimization	Determine the optimal balance between image quality and radiation or contrast dose	→ Acquire images at varying dose levels and analyze image quality metrics quantitatively and qualitatively → Multiple acquisitions to evaluate reproducibility

### Imaging system characterization

When evaluating the performance of an individual imaging system, the acquisition protocol should be designed to evaluate its behavior under different operating conditions. The goal is to understand how variations in acquisition parameters affect image quality and to ensure that the system operates optimally in a clinical environment. A step-by-step acquisition protocol for imaging system characterization is described in Table 4.

**Table 4:** Step-by-step acquisition protocol for imaging system characterization

1. Phantom preparation and positioning
a. If necessary, use modular phantoms with different test objects/modules (*e.g*., spatial resolution patterns, low-contrast inserts, etc.).
b. Position the phantom consistently in the imaging field-of-view to minimize variability.
2. Baseline (reference) acquisition
a. Perform an initial reference acquisition using manufacturer-recommended clinical settings.
b. Document all acquisition parameters.
3. Parameter variations for performance evaluation
a. Radiation dose dependence (x-ray and CT): acquire images at different tube voltage and/or exposure levels while keeping other parameters constant.
b. Energy dependence (CT and spectral imaging): evaluate the effect of varying tube voltage or energy pairings on contrast enhancement and image noise.
c. Spatial resolution and reconstruction algorithms (CT): compare images obtained with different slice thicknesses and reconstruction techniques (*e.g*., filtered back projection *versus* iterative reconstruction).
d. Temporal resolution (MRI, fluoroscopy, ultrasound): assess changes in image quality at different frame rates or repetition times.
4. Reproducibility and stability testing
a. Acquire multiple images under identical conditions to evaluate the repeatability of the measured parameter(s) and assess the stability of the imaging system. A coefficient of variation below 5% is commonly used as an acceptable threshold to assess the physical stability of imaging parameters.
b. Analyze noise fluctuations, signal stability, and image consistency.
5. Data recording and reporting
a. Maintain structured documentation of all acquisition settings.
b. Store raw data when possible for retrospective analysis.

*CT* Computed tomography, *MRI* Magnetic resonance imaging

### Comparison between imaging systems and/or technologies

Phantom studies comparing different imaging systems or technologies focus on assessing performance differences between two or more systems. Unlike individual-system characterization, these studies require rigorous standardization of acquisition settings to ensure fair comparisons. A step-by-step acquisition protocol for comparing imaging systems and/or technologies is described in Table 5.

**Table 5:** Step-by-step acquisition protocol for comparing imaging systems and/or technologies

1. Phantom preparation and positioning
a. Use the same phantom across all systems to ensure direct comparability.
b. If using an anthropomorphic phantom, confirm that internal structures and contrast elements remain stable.
2. Standardization of acquisition settings
a. Ensure equivalent settings across systems (*e.g*., matching kV_p_, mAs, slice thickness in CT), or repetition time, echo time, flip angle, bandwidth, slice thickness, sequence type in MRI.
b. If exact parameter matching is impossible, define equivalent values and acknowledge them as potential confounders.
3. Acquisition strategy
a. Perform baseline acquisitions using manufacturer-recommended clinical settings.
b. Conduct additional acquisitions with systematically adjusted parameters to allow for meaningful comparisons.
4. Repeated acquisition for statistical robustness
a. Acquire multiple images per system to account for measurement variability and ensure sufficient statistical power.
b. Use blinded observer evaluations when qualitative assessment is included.
5. Image analysis and reporting
a. Compute quantitative image quality metrics such as contrast-to-noise ratio (CNR), spatial resolution, and noise characteristics.
b. If necessary, perform a blinded subjective evaluation by radiologists to assess specific aspects of image quality (noise, contrast, and artifacts) and overall image quality.
c. Document any unavoidable differences in system design as potential sources of bias.

*CNR* Contrast-to-noise ratio, *CT* Computed tomography, *MRI* Magnetic resonance imaging

### Image quality *versus* radiation exposure or contrast dose optimization

This type of study evaluates how image quality is affected when reducing radiation dose or contrast agent concentration. The goal is to determine the lowest radiation dose or contrast level at which image quality is still maintained. A step-by-step acquisition protocol for assessing image quality in relation to radiation exposure and contrast dose optimization is described in Table 6.

**Table 6:** Step-by-step acquisition protocol for assessing image quality in relation to radiation exposure and contrast dose optimization

1. Phantom preparation and experimental setup:
a. Select a phantom with predefined contrast agent concentrations.
b. Use tissue-equivalent materials to assess radiation dose reduction.
2. Define image quality metrics:
a. Clearly define the quantitative metrics that will be used as surrogates for image quality.
b. If qualitative evaluation by multiple readers is used, clearly define the grading scales for attributes related to different aspects of image quality and/or perception (*e.g*., noise, contrast, lesion detectability, confidence in presence, etc.).
3. Systematic variation of dose or contrast levels:
a. Example 1: radiation dose optimization in CT
i. Start with a reference dose level based on standard clinical protocols.
ii. Acquire images at progressively lower dose levels while evaluating image quality.
The final goal is to identify the lowest dose at which image quality remains “diagnostically acceptable” (might be the same CNR as the reference dose level or the same overall image quality score by most readers).
b. Example 2: contrast agent reduction in CT or MRI
i. Maintain a fixed contrast agent concentration in the phantom.
ii. Adjust acquisition parameters (*e.g*., kV_p_, reconstruction algorithms) to maintain image quality despite reduced contrast dose.
iii. Test different reconstruction settings to see if increased noise reduction compensates for reduced contrast dose.
iv. Use of dual-energy or spectral CT techniques to test for material decomposition may increase contrast detectability, allowing further contrast dose reduction.
4. Repeated acquisitions for variability assessment
a. Perform multiple acquisitions per setting to ensure statistical robustness, *i.e*., precision of estimates.
b. Use predefined thresholds to define the minimum acceptable image quality.
5. Data documentation and confounding factors
a. Clearly document all acquisition settings, including unavoidable differences in system configurations.
b. Recognize and report potential confounders affecting results.

*CT* Computed tomography, *MRI* Magnetic resonance imaging

Regardless of study type, detailed documentation, consistency in phantom handling, bias minimization, and statistical robustness are essential for ensuring the integrity and reproducibility of phantom studies. Standardized acquisition protocols improve comparability across studies, enhance scientific validity, and maximize the clinical relevance of findings.

## Image quality analysis

The accuracy and robustness of image analysis methods are critical to drawing meaningful conclusions from a phantom study. The choice of analysis methods must be aligned with the study objectives, whether evaluating system performance, comparing different systems/technologies, or optimizing imaging protocols. Image quality is a general concept that encompasses many different metrics, each of which represents a specific aspect of image quality. Phantom images can be analyzed using either quantitative or qualitative methods; in either case, the method should be carefully designed to minimize bias and maximize scientific validity.

### Quantitative image quality analysis

Quantitative analysis plays a critical role in evaluating imaging system performance, comparing modalities, and optimizing acquisition protocols. This approach relies on the extraction of numerical metrics from images that provide objective, reproducible data for performance evaluation. The selection of metrics should be aligned with the study objectives and carefully defined to ensure consistency and validity across experiments.

To ensure the robustness and reliability of quantitative results, it is essential to adhere to standardized protocols for acquiring and analyzing images. This includes consistent phantom positioning, defined region-of-interest selection, and careful calibration of imaging systems to account for any variability in measurements. Furthermore, all acquisition parameters, such as exposure settings and reconstruction algorithms, should be systematically reported to allow for reproducibility and transparency in the results.

The effective use of quantitative metrics in medical imaging provides an objective means of assessing system performance, optimizing imaging protocols, and enabling meaningful comparisons across technologies. By combining basics and advanced measurements such as signal-to-noise ratio (SNR), contrast-to-noise ratio (CNR), noise power spectrum (NPS), spatial resolution (modulation transfer function (MTF)), and task-based detectability index (*d*′), researchers can capture different dimensions of image quality and its tradeoffs with factors such as radiation dose or contrast agent use. These metrics, when carefully selected and consistently applied, form the backbone of reliable imaging studies and contribute to the continuous improvement of imaging technologies and clinical outcomes.

#### SNR

SNR measures overall image clarity by comparing the intensity of the structure of interest over its noise fluctuations. SNR is a particularly important metric in modalities such as MRI and US, where maximizing the signal strength of tissues or lesions while minimizing noise is critical to diagnostic accuracy. For example, in MRI, SNR can be evaluated over different pulse sequences to determine which sequence provides the best tissue contrast while maintaining a high SNR [45, 46]. In ultrasound imaging, SNR is often used to evaluate the effectiveness of beamforming algorithms designed to reduce speckle noise and improve image clarity [47]. SNR is a critical metric when evaluating the performance of advanced imaging technologies because it directly impacts the quality of the data acquired.

It is typically calculated as follows [48]:

$${SNR}=\frac{{MPV}}{{SD}}$$

where MPV is the mean pixel value (signal) measured in a given area or detail of the phantom, and SD is the standard deviation (noise) of the pixel distributions measured in the same area.

#### CNR

One of the most frequently used metrics in medical imaging is the CNR, which quantifies the ability to distinguish a structure or lesion from the surrounding background. A higher CNR indicates better visibility of the target feature, which is particularly important in detecting low-contrast structures, such as small lesions in CT or MRI scans. In CT imaging, for example, CNR is used to evaluate the detectability of lung nodules at various radiation dose levels [49].

It is typically calculated as follows:

$${CNR}=\frac{\left|{{MPV}}_{{detail}}-{{MPV}}_{{bkg}}\right|}{{{SD}}_{{bkg}}}$$

with MPV_detail_ the MPV measured in a region of interest inside the detail one wants to calculate the CNR, MPV_bkg_ that of the surrounding background, and SD_bkg_ the noise of the background.

Factors influencing CNR include radiation dose, reconstruction algorithms, and contrast agent concentration in x-ray-based modalities, as well as pulse sequence parameters, magnetic field strength, coil design, and contrast agent concentration and properties in MRI, and transducer frequency, beamforming techniques, and gain settings in ultrasound imaging, contrast bolus timing only for dynamic or perfusion-simulating phantoms that reproduce time-dependent contrast kinetics.

To ensure reproducibility and meaningful comparisons, it is essential to consistently define regions of interest across images and perform multiple acquisitions under identical conditions to minimize variability. The consistent measurement of CNR allows for direct comparisons between imaging systems or protocols and can be used to assess the trade-offs between dose reduction and image quality. In case processed images are used, CNR is also influenced by image processing algorithms.

#### NPS

The NPS characterizes the magnitude and spatial frequency distribution of image noise, providing information beyond a single noise SD value. While SD quantifies overall noise magnitude, NPS reveals the texture or granularity of noise, which can influence both image appearance and diagnostic performance [50].

This distinction is especially important in modalities such as CT, particularly ultra-high-resolution modes, where images may have similar SD values but markedly different noise textures. In these cases, NPS enables researchers to understand whether noise is predominantly coarse or fine, which can affect the detectability of small, low-contrast features.

Factors influencing NPS include detector design, reconstruction algorithms (*e.g.*, filtered back-projection *versus* iterative), pixel size, and dose level. In phantom studies, NPS can be readily measured because repeated acquisitions under identical conditions are feasible, avoiding the variability present in clinical images.

NPS is also a fundamental input for task-based performance metrics such as the detectability index (*d*′). It is typically expressed as a function of spatial frequency and can be calculated as:

$${NPS}\left({f}_{x},{f}_{y}\right)=\frac{\Delta x\Delta y}{{N}_{x}{N}_{y}}{\left|{FFT}\left\{I\left(x.y\right)-\bar{I}\right\}\right|}^{2}$$

where $\Delta x$ and $\Delta y$ are pixel dimensions, ${N}_{x}$ and ${N}_{y}$ are the number of pixels in each direction, $I\left(x.y\right)$ is the image signal, $\bar{I}$ is the mean signal, and FFT denotes the two-dimensional Fast Fourier Transform, which converts the spatial domain representation of the noise into its frequency domain representation.

#### Spatial resolution (MTF)

Spatial resolution is a fundamental metric for determining how well an imaging system can distinguish small structures or fine details. In medical imaging, spatial resolution is typically quantified using the MTF, which describes how accurately an imaging system can reproduce variations in object contrast across different spatial frequencies. A higher MTF at a given frequency indicates better preservation of image sharpness and detail. MTF is derived from the system’s response to known high-contrast patterns and can be measured using various phantom-based methods, such as line-pair phantoms, slanted-edge targets, sharp edges, or narrow slits [51, 52]. Each approach requires specific computational techniques, for instance, edge-based methods typically involve calculating the edge spread function, which is then differentiated to yield the line spread function, from which the MTF is obtained via Fourier transformation. The resulting MTF curve describes the attenuation of contrast as spatial frequency increases, and the MTF values are typically reported in terms of spatial frequency, expressed in line pairs per millimeter (lp/mm) or line pairs per centimeter (lp/cm).

Theoretically, the higher the spatial resolution, the more finely an imaging system can resolve small features, which is essential in many clinical applications. For example, in a CT study comparing conventional and photon-counting technologies, spatial resolution analysis might show that the newer system offers improved sharpness at lower radiation doses, thus providing better image quality without compromising patient safety [53]. Higher spatial resolution is especially important in imaging applications where fine details are critical, such as mammography, where small calcifications need to be detected [54], or in CT angiography, where the detection of minute vascular structures is necessary for accurate diagnosis [55].

#### Task-based detectability index (*d*′)

The detectability index (*d*′) quantifies how well an imaging system can perform a specific detection task, such as identifying a lesion of known size and contrast, by integrating both system resolution and noise properties. Unlike SNR or CNR, which consider signal and noise separately, *d*′ incorporates the MTF and the NPS to assess how these factors jointly impact the visibility of a target.

In practice, *d*′ reflects the probability that a lesion can be distinguished from background noise by an ideal observer for a given task. This makes it highly relevant in protocol optimization, comparison of reconstruction algorithms, and evaluation of new technologies, especially when the clinical goal is the detection of subtle or small features.

Factors affecting *d*′ include task characteristics (target size, shape, and contrast), imaging system spatial resolution, noise magnitude and texture, and reconstruction method. While its calculation is more complex than CNR, it provides a more realistic estimate of performance in many diagnostic tasks.

One common form of the ideal observer detectability index is [50]:

$${d}^{{\prime} }={\left[{\int }_{u,v}\frac{{W}_{{task}}\left(u,v\right) \cdot {MTF}\left(u,v\right)}{{NPS}\left(u,v\right)}{du\; dv}\right]}^{1/2}$$

where ${W}_{{task}}$ is the Fourier representation of the target object and *u*,*v* are spatial frequency coordinates.

#### Other quantitative metrics

Many other metrics can be used, including, but not limited to, temporal resolution, dynamic range, and artifact assessment, depending on the imaging modality and study objectives. Temporal resolution is particularly critical in modalities such as fluoroscopy, digital subtraction, angiography, MRI, CT, and ultrasound, when rapid image acquisition times are necessary to capture dynamic physiological processes, including cardiac motion, vascular flow, gastrointestinal peristalsis, and urogenital tract dynamics.

### Qualitative image quality analysis

Qualitative image analysis involves the evaluation of image quality based on human perception and judgment, typically performed by trained readers (radiologists, other imaging specialists, or other professionals with an interest in particular fields of medical imaging). This subjective assessment is critical for understanding aspects of image quality that may not be captured by quantitative metrics, such as diagnostic confidence, lesion visibility, or overall image clarity [56]. While quantitative image analysis can be extremely sensitive in detecting small differences between images, these differences may not always be clinically relevant. For example, a statistically significant change in CNR may be detected without affecting the detectability of an object, making quantitative measures alone insufficient for understanding clinical relevance.

In any case, to ensure reliability and reduce bias in qualitative image analysis, it is essential to establish standardized procedures and clear grading criteria for the reader study.

#### Readersʼ selection and training

One of the most important considerations in qualitative image analysis is selecting a sufficient number of readers to reduce bias and account for variability of evaluations. Typically, more than two readers are recommended for qualitative studies, as a larger pool of readers helps reduce individual bias and improves the generalizability of results. The ideal number of readers depends on the study design and statistical power analysis, but generally ranges from 3 to 5, sometimes more than 5 readers. An odd number of readers is often used to ensure the possibility of getting an always “majority vote”. Each reader should be appropriately trained to grade the images according to predefined criteria to ensure consistency of grading.

Inter-reader reliability can be assessed using statistical measures such as κ statistics or the intraclass correlation coefficient (see below).

#### Blinding and randomization

To minimize potential bias, images should be anonymized and randomized prior to grading, ensuring that readers are blinded to the specific imaging equipment, acquisition protocol, or reconstruction parameters used. Blinding is essential to prevent any prior knowledge from influencing subjective judgments. In addition, randomizing the order of image presentation mitigates the order effect, a bias introduced when the sequence of image review influences perception and scoring.

#### Evaluation criteria and scales

Clear and precise definitions of grading criteria are essential to ensure that readers rate images consistently. Typical criteria include noise level, contrast visibility, lesion detectability, and artifact presence, each of which can affect the perceived quality of an image. Noise levels are often evaluated based on how grainy or smooth the image appears. Higher noise can degrade image quality and make structures harder to see. Contrast visibility refers to the ability to distinguish different tissue types or structures, such as lesions, from the background. Lesion detectability evaluates the ability to identify abnormal structures, such as tumors or nodules, and may include specific assessments of size, location, and clarity. Artifacts are distortions or abnormalities introduced by the imaging process, not related to local characteristics of the phantom, that can obscure important details or create false findings.

Absolute Likert scales or similar ordinal rating systems are commonly employed to rate image quality attributes, using predefined levels (*e.g*., 1–5 or 1–7) to represent gradations of quality. For example, a 1–5 Likert scale might represent a continuum from “poor” (1) to “excellent” [56]. For the sake of reproducibility, it is important to define as accurately as possible each step of the scale to avoid ambiguity. For example:

- 1 (poor) = severe image degradation with noticeable artifacts or marked noise that interferes with lesion detection;
- 2 (fair) = some visible noise or artifacts, but lesions can still be detected with some difficulty;
- 3 (good) = adequate image quality, minor noise/artifacts that do not markedly affect lesion visibility;
- 4 (very good) = high quality image with minimal noise and artifacts, lesions are easily identifiable;
- 5 (excellent) = optimal image quality, clear and detailed structures without noticeable noise or artifacts.

Each reader independently scores the images based on these predefined criteria, and their scores are recorded for analysis.

An alternative to absolute Likert scales are relative Likert scales, which present pairs of images/studies side by side and ask readers to rate them in comparison to each other [57]. This approach aims to assess differences, rather than assigning absolute ratings. It is particularly effective when the goal is to highlight differences between imaging conditions, systems, or acquisition settings.

A relative Likert scale typically ranges from -2 to +2, with 0 representing no difference or equal conditions between the systems or protocols being compared. The scale is used to measure how one system or modality compares to another on specific attributes, with negative values indicating that the first system performs worse and positive values indicating that it performs better on the evaluated aspect. For example:

- +2 = the first system or condition is markedly better than the second system in terms of image quality (*e.g*., better visibility, reduced noise, improved contrast);
- +1 = the first system or condition is slightly better than the second, but the difference is minimal and may not significantly affect clinical interpretation;
- 0 = both systems or conditions are equal in image quality; there is no noticeable difference:
-1 = the first system or condition is slightly inferior to the second, with a slight disadvantage in image quality.
-2 = the first system or condition is markedly worse than the second, with noticeable negative effects on image quality (*e.g*., higher noise, poor contrast, or lower resolution).

## Sample size calculation, data presentation, and statistical analysis

Sample size calculation is frequently omitted in phantom studies. As per any experiments, this may be a cause of data paucity and lack of statistical power. We refer here to the number of repeated measurements for each modality, technique, or protocol, but also to the number of readers or any other variables that can be expanded in a phantom study without the ethical issues typical of clinical studies. When possible, the potential variability of the same model of phantom can also be considered [41]. There are plenty of resources for calculating the sample size. A suggestion is to follow the recent recommendations by Monti et al [22]. Importantly, these calculations oblige a clear definition of primary, secondary, and exploratory endpoints.

When reporting the results of a phantom study, clarity, structure, and transparency are essential to enable accurate interpretation and facilitate replication of the experiment. Providing sufficient detail ensures that other researchers can assess the robustness of the analysis, critically evaluate the results, and, if necessary, replicate the study. The results should be presented in a manner that is consistent with the study endpoints and emphasizes the technological or clinical implications.

Quantitative variables: the key components to properly describe the distribution of the data, are reporting mean and SD together with median and interquartile range, allowing the reader to evaluate if the data are well described by a normal or near-normal distribution or by skewed or non-normally distributed data.

Qualitative assessments and group comparison: especially when using Likert scales or other subjective assessments, it is useful to report the frequency distribution of ratings, with percentages for each category or scale level; in addition, include measures of central tendency (median or mode) to provide insight into the overall trend of responses [58]. To compare Likert scale data across groups (*e.g*., comparing ratings from different imaging modalities, protocols, or postprocessing techniques), the use of nonparametric statistical tests such as Mann–Whitney *U*-test for independent samples and Wilcoxon signed-rank test for paired samples is encouraged [58, 59]; the use of a parametric tests is in general admitted [60], although in this setting care must be used and conditions for their application must be checked [61].

For all estimates, provide 95% confidence intervals around the point estimate to convey the precision of the results.

Inter-rater agreement: if more than one reader is involved, report inter-rater reliability metrics. For categorical data, use Cohen κ (for two readers, weighted κ in the case of ordinal measurement such as Likert scales) or Fleiss κ (for three or more readers, or an extension valid also for ordinal data [62]); for continuous data, use the intraclass correlation coefficient [63].

### Sensitivity analysis

Sensitivity analysis is a valuable tool for exploring how variations in input parameters influence the outcomes of a model or experimental study. It addresses the fundamental question: “If I change one factor, how does this affect the results?” [64]. In the context of image quality assessment, in particular when using absolute or relative Likert scales, sensitivity analysis can help determine whether small changes in the interpretation of rating categories (*e.g*., “neutral” or “equal”) materially impact the study conclusions. This type of analysis is especially recommended in studies with small sample sizes, subjective scoring systems, or where critical results are dependent on specific thresholds (*e.g*., dichotomizing Likert scores). In such cases, results may be highly sensitive to assumptions about scale interpretation. Conversely, in large-sample studies using well-established scoring systems and well-defined endpoints, sensitivity analysis may be less critical because individual variations in scoring are unlikely to affect the overall findings.

### Outliers, tests, and *p*-values

Any data exclusions (*e.g*., outlier removal) should be clearly stated and justified. The rationale for choosing statistical tests should be provided. Each comparison and the corresponding statistical tests applied must be listed in the Statistical analysis subsection of the “Methods” section. In addition, the results of any statistical tests performed must be reported, providing exact *p-*values (not *p* < 0.05 or *p* ≥ 0.05).

### Tables and figures

Present quantitative and qualitative data in clearly organized tables or figures. Graphs, such as bar graphs or box plots, can be used to visually compare key metrics across experimental conditions. Tables should provide a detailed breakdown of results by condition, acquisition settings, or imaging systems to ensure that all relevant details are easily accessible.

### Transparency

Ensure transparency by clearly describing any deviations from the study protocol, variations in acquisition settings, or technical problems encountered during the study. Providing this information allows readers to assess potential sources of bias, data variability, or limitations in the experimental design, and ensures that the results are interpreted in the proper context.

## Building your discussion section

As for clinical studies, the discussion section of a phantom study is where the results are interpreted, contextualized, and compared to the existing literature. This section should clearly highlight the contributions of the study, acknowledge its limitations, and suggest potential technological or clinical implications.

### Interpretation of your results

First, link the findings to the study objectives. Begin by reviewing the study objectives and summarizing how the results address these objectives. For example, if the goal of the study was to evaluate the effect of dose reduction on image quality, summarize whether the results demonstrate that dose reduction was achieved without compromising critical quality metrics. This will help readers understand how the results contribute to answering the research questions. Particular aspects, including technical issues or innovative approaches, can be briefly highlighted and discussed.

Second, present a comparison to previous similar phantom or clinical studies. Highlight both agreement and disagreement with the literature. If your results are consistent with previous studies, discuss the reasons for this agreement. Conversely, if there are differences, explore potential factors such as variations in phantom design, acquisition protocols, imaging technology, or statistical methods. This comparison helps contextualize the study within the broader research landscape and lends credibility to the results.

Third, discuss technical and/or clinical implications, such as the reduction in energy consumption in CT or the need for further technical refinement of a new MRI sequence. Importantly, describe the potential clinical significance of your results. For example, dose reduction or scanning time without sacrificing image quality. Link the phantom study results to real-world clinical scenarios to help readers understand how the study results could impact patient care or clinical workflow.

### Limitations of your study

As for clinical studies, it is important to recognize the inherent limitations of any phantom study design. For phantom studies, there is an additional inherent limitation: phantoms are simplified representations of human tissues and anatomy. They may never fully replicate real-world clinical conditions.

Thus, first discuss how much the phantom model used in the study accurately represented the tissue types and imaging conditions encountered in practice. Were any important anatomical features or disease conditions not included or underrepresented?

Second, address potential challenges related to the reproducibility of the study results in the clinical setting. Variations in patient anatomy, clinical protocols, or imaging systems may affect how results translate to real-world practice. Be aware that the controlled conditions of the phantom study may differ from the dynamic and variable nature of the clinical environment.

Third, consider any limitations in the image quality metrics used and the statistical analysis performed. For example, a small sample size or lack of statistical power may limit the reliability of the findings. If biases in data collection, measurement techniques, or statistical approaches may have influenced the results, these should be discussed openly to ensure a balanced interpretation.

### Recommendations for future research

Based on the findings and limitations identified in your study, suggest directions for future research. These could include the following points.

- Improved phantom models: propose research into more complex or anatomically accurate phantoms that better mimic human anatomy, pathological conditions, or clinical variability. Such phantoms could provide more realistic insights into imaging performance under conditions closer to real-world practice.
- Alternative technologies and protocols: recommend the investigation of alternative imaging technologies, techniques, or acquisition protocols that could improve image quality, reduce radiation dose, or optimize diagnostic accuracy. Identifying promising avenues for future advances can guide ongoing innovation in medical imaging.
- Clinical validation: encourage the design of clinical studies that validate phantom study results with real patient data. Emphasize the importance of testing the impact of new imaging systems or protocols on patient safety, diagnostic accuracy, and workflow in clinical settings.

## Your conclusions

Conclude the discussion by summarizing the key findings of the study and their practical implications. Reiterate the importance of the study in advancing scientific knowledge or improving clinical practice. Emphasize how the results may contribute to the development of better imaging technologies, improved patient care, or optimized imaging protocols. Finally, outline the broader implications of the findings for the field of medical imaging and future research efforts.

## The PSMI checklist

To facilitate the authors in trying to adhere to the many recommendations contained in this paper, we propose a “PSMI“ checklist composed of 13 sections and 25 items (Table 7). Authors can fill in the PSMI checklist and add it as an attached document to any submission of phantom studies to this journal or other biomedical journals, making the editors’ and reviewers’ jobs easier in assessing the quality of the submitted manuscript.

**Table 7:** The 13-section 25-item PSMI checklist

Section/Topic	Item to report	☑	Page
Title	1. Clearly indicate that the study is a phantom-based study		
Abstract	2. Briefly describe the phantom type in the methods		
Background/Introduction	3. State the phantom-based research hypotheses and objectives		
Methods: study design	4. Specify the study type (*e.g*., technology validation, system performance comparison, protocol optimization, quality control)		
Methods: phantom description	5. Describe the type of phantom (standard, anthropomorphic, mixed, bio-phantom, computational) and materials used		
	6. In the case of a custom-made phantom, fully describe the fabrication and preparation process		
	7. Justify the phantom selection based on study objectives		
Methods: imaging acquisition protocol	8. Provide full details of acquisition parameters, phantom setup, and number of acquisitions		
	9. Standardize acquisition settings across systems/technologies when comparing		
Methods: image analysis	10. Describe quantitative analysis metrics (*e.g*., signal- or CNR) and how they were measured, including region-of-interest placement strategy		
	11. If included, describe qualitative assessment: criteria, number of readers, blinding, and rating scale(s) used		
Methods: statistics	12. Make a preliminary calculation of sample size		
	13. Specify statistical tests and justify their selection		
	14. Report how intra-condition variability (repeated acquisitions) was handled		
	15. Describe inter-reader agreement analysis if applicable (*e.g*., κ statistics, intraclass correlation coefficient)		
Results: quantitative analysis	16. Report means and SDs or medians and interquartile ranges, depending on data distribution		
	17. Present the results of statistical comparisons with *p*-values, effect sizes, and 95% confidence intervals		
Results: qualitative analysis	18. Report reader assessments and inter-reader agreement		
Results: tables and figures	19. Provide clear and organized tables and/or figures for quantitative and qualitative data		
Discussion	20. Summarize main results		
	21. Compare findings with existing literature, if available		
	22. Discuss technological and/or clinical implications		
	23. Acknowledge study limitations (*e.g*., phantom model, reproducibility, statistical power)		
Conclusions	24. Summarize key findings and their significance for imaging practice or research.		
	25. Suggest directions for future research		

*CNR* Contrast-to-noise ratio

## The connection to clinical implications

PSMI enables controlled, reproducible experiments that would be impractical, unethical, or prohibitively expensive to conduct in humans or animal models. They allow researchers to isolate and assess specific technical parameters, evaluate system performance, and optimize protocols in a cost- and time-efficient manner. However, even in the context of purely technical investigations, it is essential to maintain a clear connection to the clinical implications of the findings. Importantly, the absence of a positive finding in a phantom study should not be undervalued. A well-designed “negative” study can offer critical insights, such as demonstrating the limited clinical utility of a proposed innovation, and help redirect resources and research efforts more effectively.

Professionals involved in phantom studies must recognize that image analysis alone does not establish the clinical relevance of a technological innovation. The development of 3-T MRI illustrates this point: for many years, research focused primarily on demonstrating superior image quality compared to 1.5-T systems, without providing evidence of improved diagnostic accuracy or clinical benefit [65, 66]. This underscores a key principle: in medical imaging, the ultimate objective is not to perfect images or phantoms, but to improve patient care. Maintaining a clinical perspective ensures that phantom-based investigations contribute meaningfully to translational progress and medical decision-making.

## Abbreviations

${d}^{{\prime} }$: Task-based detectability index
3D: Three-dimensional
AI: Artificial intelligence
CNR: Contrast-to-noise ratio
CT: Computed tomography
MPV: Mean pixel value
MRI: Magnetic resonance imaging
MTF: Modulation transfer function
NPS: Noise power spectrum
PSMI: Phantom studies in medical imaging
SD: Standard deviation
SNR: Signal-to-noise ratio

## Footnotes

1 We refer here to protons of ^1^H nuclei, mainly from water and lipids in clinical MRI.
